# Genetic Modifiers of MeCP2 Function in *Drosophila*

**DOI:** 10.1371/journal.pgen.1000179

**Published:** 2008-09-05

**Authors:** Holly N. Cukier, Alma M. Perez, Ann L. Collins, Zhaolan Zhou, Huda Y. Zoghbi, Juan Botas

**Affiliations:** 1 Department of Molecular and Human Genetics, Baylor College of Medicine, Houston, Texas, United States of America; 2 Neurobiology Program, Children's Hospital Boston, Massachusetts, United States of America; 3 Departments of Neurology and Neurobiology, Harvard Medical School, Boston, Massachusetts, United States of America; 4 Departments of Neuroscience and Pediatrics, Baylor College of Medicine, Houston, Texas, United States of America; 5 Howard Hughes Medical Institute, Baylor College of Medicine, Houston, Texas, United States of America; 6 Department of Molecular and Cellular Biology, Baylor College of Medicine, Houston, Texas, United States of America; University of Minnesota, United States of America

## Abstract

The levels of methyl-CpG–binding protein 2 (MeCP2) are critical for normal post-natal development and function of the nervous system. Loss of function of MeCP2, a transcriptional regulator involved in chromatin remodeling, causes classic Rett syndrome (RTT) as well as other related conditions characterized by autism, learning disabilities, or mental retardation. Increased dosage of MeCP2 also leads to clinically similar neurological disorders and mental retardation. To identify molecular mechanisms capable of compensating for altered MeCP2 levels, we generated transgenic *Drosophila* overexpressing human MeCP2. We find that MeCP2 associates with chromatin and is phosphorylated at serine 423 in *Drosophila*, as is found in mammals. MeCP2 overexpression leads to anatomical (i.e., disorganized eyes, ectopic wing veins) and behavioral (i.e., motor dysfunction) abnormalities. We used a candidate gene approach to identify genes that are able to compensate for abnormal phenotypes caused by MeCP2 increased activity. These genetic modifiers include other chromatin remodeling genes (*Additional sex combs*, *corto*, *osa*, *Sex combs on midleg*, and *trithorax*), the kinase *tricornered*, the UBE3A target *pebble*, and *Drosophila* homologues of the MeCP2 physical interactors Sin3a, REST, and N-CoR. These findings demonstrate that anatomical and behavioral phenotypes caused by MeCP2 activity can be ameliorated by altering other factors that might be more amenable to manipulation than MeCP2 itself.

## Author Summary

Rett syndrome (RTT) is a progressive neurodevelopmental disorder that affects girls early in childhood and is caused by mutations in the *MECP2* gene. Loss of MeCP2 function can also lead to clinically distinct conditions characterized by autism, learning disability, and mental retardation. Remarkably, increased levels of MeCP2 leads to related neurological disorders and mental retardation as well. These data emphasize the critical importance of regulating MeCP2 protein levels for normal post-natal development and function of the nervous system. MeCP2 is a protein that associates with chromatin and is thought to modulate gene expression. We have generated *Drosophila* that overexpress human MeCP2 to investigate the possibility that adjusting the activity of other genes may compensate for altered levels of MeCP2. In support of this hypothesis, we found a variety of modifier genes, including chromatin remodeling genes, that are able to ameliorate and/or aggravate the consequences of MeCP2 overexpression. These findings open the possibility of therapeutic avenues for RTT and related neuropsychiatric disorders by targeting proteins that are possibly easier to manipulate than MeCP2 itself.

## Introduction

Research in the last decade has linked the methyl-CpG-binding protein 2 (MeCP2) with a variety of related neurological disorders [1]. Loss of MeCP2 function causes classic Rett syndrome (RTT), but can also lead to related neurological conditions with symptoms that include autism, mild or severe mental retardation with seizures, or learning disabilities [2],[3]. Increased dosage of the *MECP2* locus also leads to RTT-like features and severe mental retardation [4]–[6]. Similar phenotypes are recapitulated in mice that either lack or overexpress *MECP2*, thus underscoring the importance of properly regulating MeCP2 levels [7]–[10]. The MeCP2 protein contains a methyl-CpG-binding domain (MBD) and localizes to the heterochromatin where it is believed to regulate gene expression by recruiting histone deacetylases to alter chromatin structure [11],[12]. While an ortholog for the complete MeCP2 protein does not exist in *Drosophila*, methyl-CpG-binding domains are conserved from flies to humans [13]. MeCP2 also interacts with other proteins involved in transcriptional repression and chromatin remodeling including Sin3a, REST and Brahma, a core component of the SWI/SNF complex [14]–[16]. These and other previously identified MeCP2 interactors have well conserved orthologs in *Drosophila* (Table S1), as do most components of the chromatin remodeling machinery. Examples of evolutionarily conserved chromatin remodeling proteins include members of the Polycomb and trithorax groups (Pc-G and trx-G, respectively), as well as proteins involved in histone tail modification [17],[18]. Many of these proteins act in protein complexes that function antagonistically to promote either activation or repression of target genes [17], [19]–[21]. Therefore, we hypothesized that genetic screening in transgenic flies expressing human MeCP2 may permit the identification of genes capable of compensating the phenotypes caused by altered MeCP2 levels. These genetic modifiers may include genes that function antagonistically to MeCP2 in chromatin remodeling, and perhaps other genes modulating MeCP2 functions or interactions. Here we report the identification of such genes.

## Results

### Expression of Human MeCP2 in *Drosophila*

**Figure 1:**
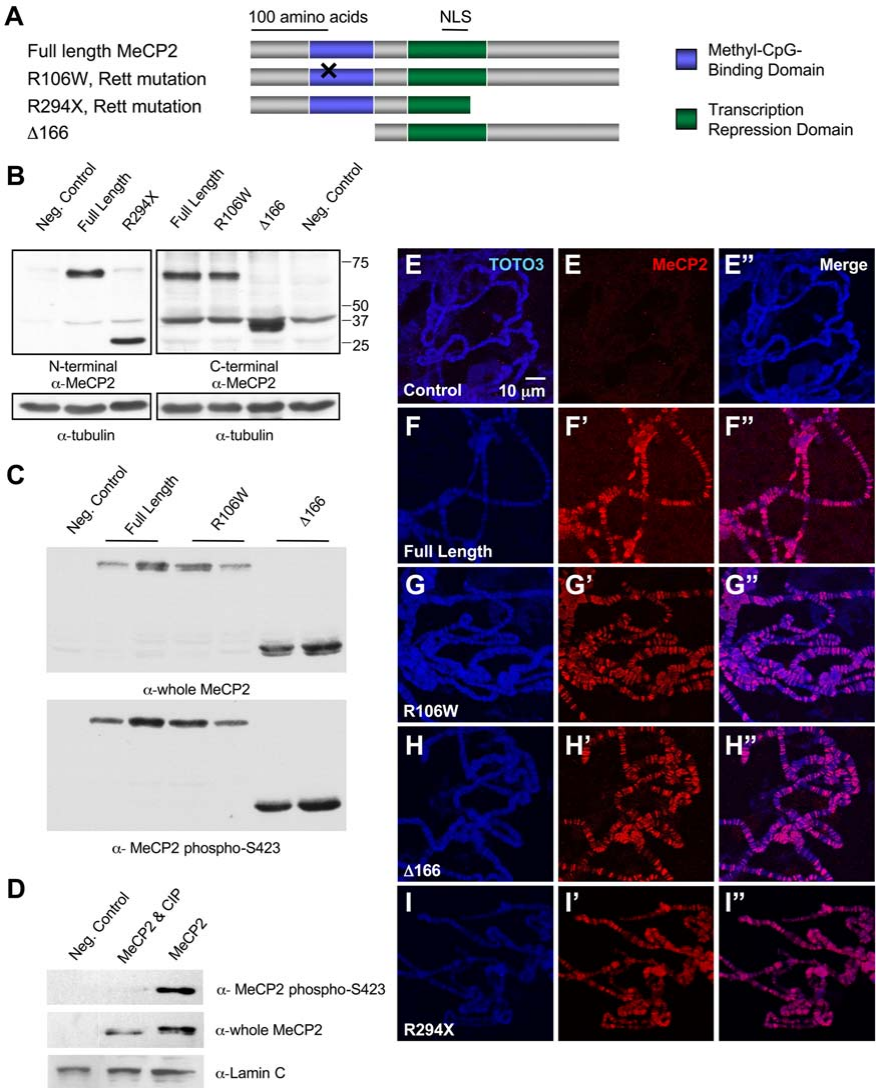
MeCP2 alleles used to generate transgenic *Drosophila*: protein expression, phosphorylation at serine 423, and association with polytene chromosomes. A. Four MECP2 alleles were cloned into pUAST to generate transgenic flies. The methyl-CpG-binding domain (MBD) is represented by blue boxes and the transcription repression domain (TRD) is represented by green boxes. The nuclear localization signal (NLS) falls within the TRD. B. Western blot analysis demonstrates expression of each of the alleles when driven by *GMR-Gal4*. Two distinct MeCP2 antibodies were utilized in order to recognize each allele to confirm that a deletion removed an epitope region. C. Immunoblot with a phospho-specific antibody shows phosphorylation in the three alleles retaining amino acid S423. D. Immunoblot with the phospho-specific MeCP2 S423 antibody in negative control, extracts from MeCP2 expressing flies when treated with calf intestinal phosphatase and untreated MeCP2 extracts. The treated samples fail to produce a band with the phospho-specific antibody, but demonstrate MeCP2 expression with the whole MeCP2 antibody (E-I”). Immunoflourescence of squashed polytene chromosomes dissected from 3rd instar larvae raised at 25°C. Control larvae do not have MeCP2 immunoreactivity (E-E”). All MeCP2 alleles demonstrate accumulation of the MeCP2 protein in banded pattern along the polytene chromosomes (F-I”).

We generated transgenic flies overexpressing wild-type MeCP2 as well as three mutant alleles using the human *MECP2_e2* cDNA (Figure 1A). The RTT R106W allele produces a missense mutation within the MBD that eliminates the protein's ability to bind DNA [22]. The RTT R294X mutation truncates the protein within the transcriptional repression domain (TRD), but maintains the nuclear localization signal. The Δ166 allele completely removes the MBD and N-terminal portion of the protein. Constructs were created inserting each allele into the pUAST vector to utilize the GAL4-UAS system [23]. This system controls expression in specific cell types depending on the Gal4 driver line used, and can be modified by varying the temperature of the fly cultures – increased temperature leads to increased expression.

Independent MeCP2 transgenic lines of each allele were generated and tested to ensure that any resulting phenotypes were not caused by the insertion site. Using *GMR-Gal4* to drive transgene expression in the eye [24], we confirmed protein expression by western blot analysis using extracts from whole fly heads (Figure 1B). Furthermore, we found that all three MeCP2 alleles that retain amino acid S423, which corresponds to murine S421, produce protein that is specifically phosphorylated at this site (Figure 1C). This specific signal was abolished when the protein extract was treated with alkaline phosphatase (Figure 1D). Phosphorylation at this serine in mammals is brain specific, and it is required by MeCP2 to control dendritic patterning, spine morphogenesis and to regulate the BDNF target gene [25]. Therefore, this key posttranslational modification is conserved when MeCP2 is expressed in *Drosophila*.

Association of MeCP2 with chromatin is a functional property of the mammalian protein [12], and was evaluated by promoting MeCP2 expression with the ubiquitous *Actin5c-Gal4* driver. Immunofluorescent staining of squashed salivary glands demonstrated that MeCP2 localizes to the nucleus and associates with polytene chromosomes along many bands in all four alleles (Figure 1E-I”). While there is widespread association, MeCP2 does not localize to all polytene bands, suggesting target specificity. Association with the polytene chromosomes does not solely depend upon the MBD or C-terminal regions since all four proteins behave similarly. The ability of the R106W and Δ166 mutants to associate with chromosomes implies that the methyl-CpG-binding domain is not required for this activity. Additional factors, possibly functioning in various protein complexes with MeCP2, may act to recruit MeCP2 to the chromatin.

### Phenotypes Caused by MeCP2 Overexpression in *Drosophila*

**Figure 2:**
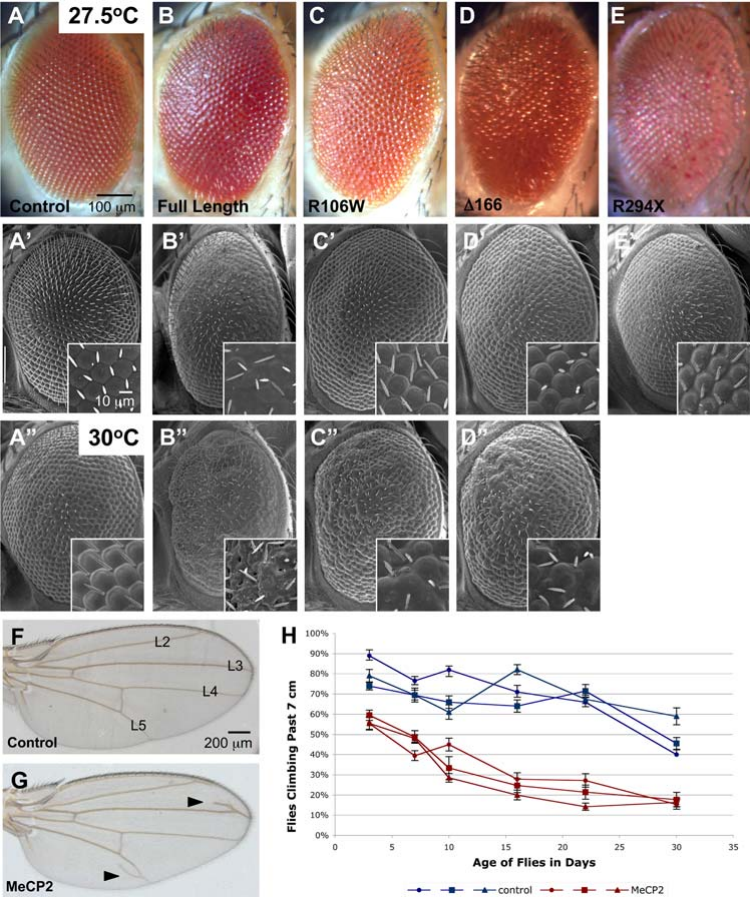
MeCP2 overexpression leads to eye, wing and motor performance phenotypes. Light microscope images (A–E), and scanning electron microscope images (A'–D”) of fly eyes from controls or animals expressing MeCP2 driven by *GMR-Gal4* driver at either 27.5°C or 30°C. External eyes of control flies show normal ommatidial organization, while eyes from animals expressing any of four distinct MeCP2 alleles show disruption in the structured pattern of the eye the surface. Note increased severity of the phenotypes at the higher temperature. F–G. The *C5-Gal4* driver was used to drive either UAS-lacZ or full-length MeCP2 throughout the wing pouch at 25°C. Compared to controls, MeCP2 expressing flies have extra vein tissue (arrowheads) near L3 and L5. H. The neuronal driver *CHA-Gal4* was used to drive expression of either UAS-eGFP or full-length MeCP2 at 25°C. Each sample represents a group of 20 virgin females. Beginning at 3 days of age, a lower percent of MeCP2 expressing flies are able to climb to 7 cm in 18 seconds as compared to control flies (Repeated measures ANOVA p<0.001). Over time, both groups decrease in their ability to climb. Error bars represent the standard error. Genotypes: A-A”, *GMR-Gal4*/+. B-B”, *GMR-Gal4:UAS-MeCP2^FLM119-2M^*/+. C-C”, *GMR-Gal4:UAS-MeCP2 R106W/+.* D-D”, *GMR-Gal4:UAS-MeCP2 Δ166/+*. E-E', *GMR-Gal4*:*UAS-MeCP2 R294X/+.* F, *C5-Gal4*/+. G, *C5-Gal4*:*UAS-MeCP2^ FLM119-1M^*/+. H, *CHA-Gal4/UAS-eGFP* and *CHA-Gal4*/*UAS-MeCP2^ FLM119-2M^.*

MeCP2 overexpression in the fly eye by *GMR-Gal4* was utilized as an assay for rapid genetic screening of modifiers. Overexpression of multiple independent lines of all four alleles resulted in external eye phenotypes of varying degrees (Figure 2A–E). Lines expressing comparable protein levels were selected for each allele (Figure 1B). The full-length wild-type, R106W and Δ166 lines cause a disruption of the external structure of the eye that is recognized as a “glassy” effect on the surface when observed by light microscopy (Figure 2A–D). When evaluated by scanning electron microscopy, these same animals show disorganized ommatidia and partial loss of interommatidial bristles (Figure 2A'–D'). These features were enhanced in flies cultured at a higher temperature (Figure 2A”–D”) as a result of elevated expression levels. Of all four alleles, the full-length protein causes the strongest disruption to the external eye. While the R294X allele does not cause an obvious disruption of the external eye structure, it shows a loss of pigmentation phenotype (Figure 2E, 2E', and data not shown), which had only been seen in one of the most strongly expressing full-length lines. Moreover, expression of the R294X allele at a higher temperature is lethal, possibly a consequence of the leaky expression of the *GMR-Gal4* driver into other tissues.

We also overexpressed MeCP2 in other fly tissues. Expression of the full-length protein in the wing pouch by *C5-Gal4* produces extra vein tissue around the L3 and L5 wing veins (Figure 2F, G). Furthermore, neuronal expression of full-length MeCP2 by the *CHA-Gal4* driver [26] leads to impaired motor function in adult flies as measured in a climbing assay (Figure 2H, Video S1). While external eye phenotypes are most practical for primary screening to identify novel genetic modifiers of MeCP2, both the wing vein and climbing phenotypes are valuable as secondary screening assays to validate genetic interactions.

### Genetic Modifiers of MeCP2

**Figure 3:**
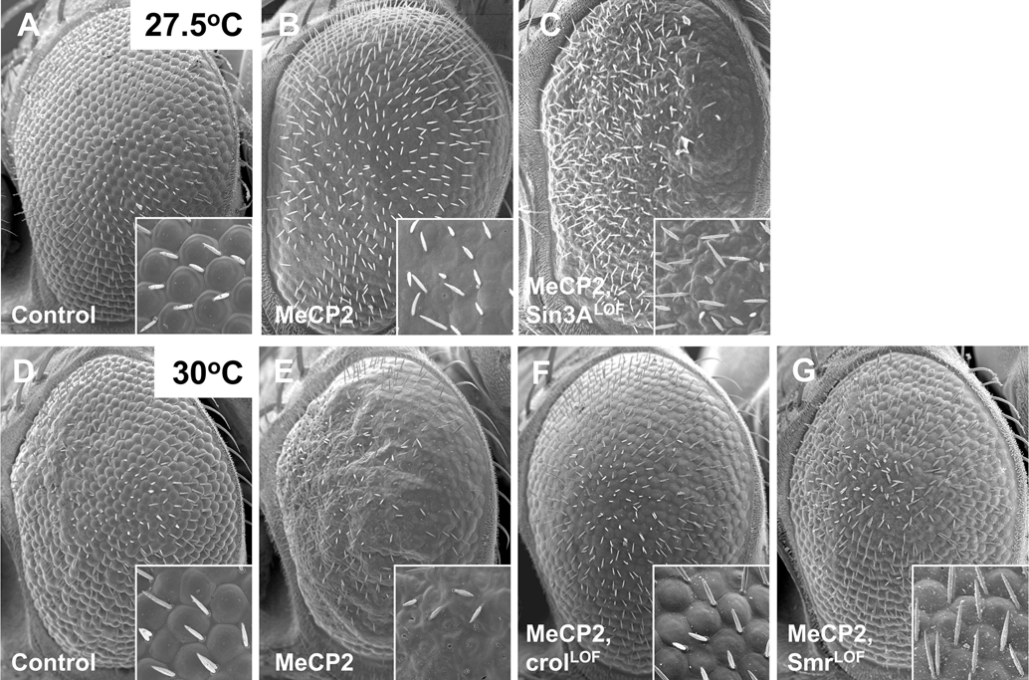
Known MeCP2 physical interactors are also genetic modifiers of the MeCP2 eye phenotype. A–C. SEM images of the external eye of a control, a fly expressing full-length MeCP2, and full-length MeCP2 in the presence of a heterozygous loss-of-function allele of Sin3A cultured at 27.5°C. Reduced Sin3A activity enhances the disorganization of the ommatidia in the eye. D–G. SEM images of the external eye of a control, a fly expressing full-length MeCP2, and full-length MeCP2 in the presence of either loss-of-function of crooked legs or Smrter cultured at 30°C. Both alleles suppress the ommatidial disorganization caused by MeCP2 expression in the eye. Genotypes: A. *GMR-Gal4/+.* B, *GMR-Gal4:UAS-MeCP2^ FLM119-1M^/+.* C, *GMR-Gal4:UAS-MeCP2^ FLM119-1M^/Sin3A^dQ4^.* D, *GMR-Gal4/+.* E, *GMR-Gal4:UAS-MeCP2^ FLM119-2M^/+.* F, *GMR-Gal4:UAS-MeCP2^ FLM119-2M^/crol^d03416^.* G, *Smr^e04389^/+; GMR-Gal4:UAS-MeCP2^ FLM119-2M^/+*.

We rationalized that *in vivo* genetic modifiers of MeCP2 function might be enriched among known MeCP2 physical interactors. In support of this hypothesis we previously showed that a large proportion of the physical interactors of huntingtin (the protein that when mutant causes Huntington's disease) are also genetic modifiers of huntingtin-induced neurodegeneration [27]. To test this hypothesis in the case of MeCP2, we evaluated *Sin3A*, *Smrter*, and *crooked legs*, the *Drosophila* homologs of *Sin3a*, *N-CoR*, and *REST* (Table S1) [28],[29]. We found that heterozygous loss-of-function mutations in each of these three direct interacting partners alter the MeCP2 eye phenotype (Table 1, Figure 3).

**Table 1:** Genetic Modifiers of MeCP2.

		MeCP2 alleles		
*Drosophila* Gene, Flybase ID Mammalian Homolog(s)	Modifier Allele	Full length	Δ166	R294X
***crooked legs (crol),*** ** FBgn0020309**	c04670 (LOF)	sup	sup	sup
*RE1 silencing transcription factor (REST)*	e0407 (LOF)	sup	sup	sup
***Sin3a,*** ** FBgn0022764**	dQ4 (LOF)	enh	enh	sup
*Sin3a*				
***Smrter (Smr),*** ** FBgn0024308**	e04377 (LOF)	sup	sup	sup
*nuclear receptor co-repressor (N-CoR)*	e04389 (LOF)	sup	sup	no mod
***Additional sex combs (Asx)***	1 (GOF)	enh	enh	enh
**FBgn0000141**	EY07384 (LOF)	sup	sup	sup
*Additional Sex Combs like 1(Asxl1)*	XF23(LOF)	sup	sup	sup
***corto,*** ** FBgn0010313**	07128b (LOF)	sup	sup	sup
*Mastermind like 2 (Maml2)*	c03244 (LOF)	sup	sup	sup
	e02822 (LOF)	sup	sup	sup
***osa,*** ** FBgn0003013**	00090 (LOF)	sup	sup	sup
*AT-rich interaction domain 1a & 1b*	308 (LOF)	sup	sup	sup
*(Arid1a, Arid1b)*	EY09619 (LOF)	sup	no mod	sup
	UAS-osa (OE)	enh	enh	enh
***pebble (pbl),*** ** FBgn0003041**	2 (LOF)	sup	no mod	sup
*Epithelial cell transforming*	3 (LOF)	sup	no mod	sup
*sequence 2(Ect2)*	5 (LOF)	sup	no mod	sup
	09645 (LOF)	sup	no mod	no mod
	UAS-pebble (OE)	enh	enh	enh
***Sex combs on midleg (Scm)***	e01989 (LOF)	sup	sup	no mod
**FBgn0003334**	D1 (LOF)	sup	no mod	sup
*Sex combs on midleg homolog 1(Scmh1)*	ET50e (LOF)	sup	sup	no mod
**	M36 (LOF)	sup	sup	sup
	M56 (LOF)	sup	sup	sup
	UAS-scm (OE)	enh	enh	no mod
	XF24 (LOF)	sup	sup	sup
***tricornered (trc),*** ** FBgn0003744**	UAS-trc LD (OE)	sup	sup	sup
*Nuclear Dbf-related 1 & 2 (Ndr1, Ndr2)*	UAS-trc wtn (OE)	sup	sup	sup
***trithorax (trx),*** ** FBgn0003862**	1 (hypomorph)	enh	enh	no mod
*Mixed lineage leukemia (Mll)*	E2 (amorph)	enh	enh	sup
	KG04195 (LOF)	enh	enh	no mod

LOF: loss of function, GOF: gain of function, OE: overexpression.

no mod: no clear modification with this particular allele.

We then tested other candidate modifier genes that were chosen based on their functions. In addition to chromatin remodeling genes, these included a collection of kinases because MeCP2 is phosphorylated [25], and two genes implicated in Angelman syndrome, a disorder that shares clinical features with Rett syndrome. These last two candidates are the *Drosophila* homolog of *UBE3A*, the gene encoding a ubiquitin ligase misregulated in Angelman syndrome, and its target *pebble* [30],[31]. When available, both loss-of-function and overexpression mutant *Drosophila* lines of each candidate were collected. A total of 584 mutant *Drosophila* lines were obtained and screened against the full-length MeCP2 allele; 392 lines representing 158 individual kinases, 174 lines representing 54 unique chromatin remodeling genes, and 18 lines encompassing *UBE3A* and *pebble* mutants.

**Figure 4:**
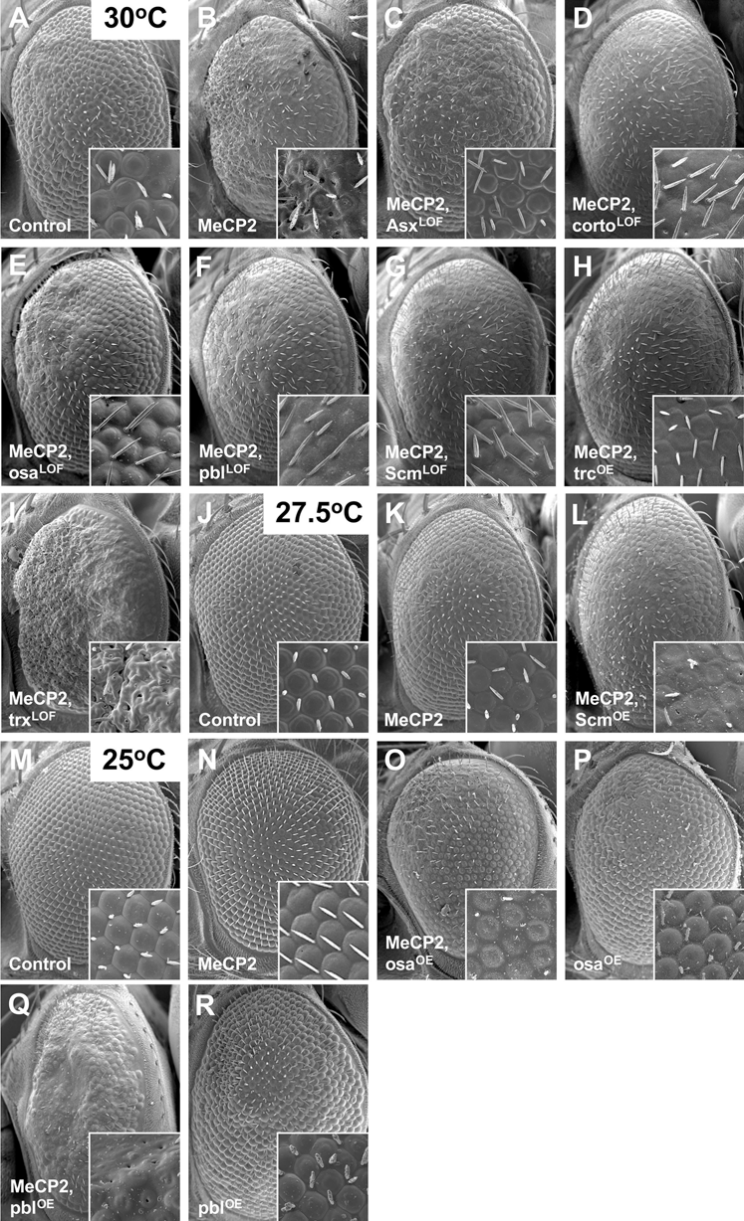
Novel genetic modifiers of the MeCP2 eye phenotype. A–B. MeCP2 expression by *GMR-Gal4* at 30°C causes severe disorganization of the ommatidia and interommatidial bristles compared to controls. C–H. This phenotype is alleviated when combined with loss-of-function mutants in Asx, corto, osa, pbl, Scm, or overexpression of trc. I. In contrast, the loss-of-function trx allele enhances the external eye phenotype. J–L. When MeCP2 is driven by *GMR-Gal4* at 27.5°C, the mild eye phenotype of MeCP2 is enhanced when combined with overexpression of Scm. M–R. Even though the MeCP2 flies do not show an eye phenotype at 25°C (N), when combined with either overexpression of osa (O) or pbl (Q), a strongly disrupted phenotype results that causes a loss of interommatidial bristles and, in the case of pbl, a reduction in the number of ommatidia. When osa and pbl are overexpressed alone, they have very mild phenotypes (P, R). Genotypes: A, *GMR-Gal4/+.* B, *GMR-Gal4:UAS-MeCP2^FLM119-2M^/+.* C, *GMR-Gal4:UAS-MeCP2^FLM119-2M^/Asx^XF23^.* D, *GMR-Gal4:UAS-MeCP2^FLM119-2M^/+; corto^c03244^/+.* E, *GMR-Gal4:UAS-MeCP2^FLM119-2M^/+; osa^00090^/+.* F, *GMR-Gal4:UAS-MeCP2^FLM119-2M^/+; pbl^09645^/+.* G, *GMR-Gal4:UAS-MeCP2^FLM119-2M^/+; Scm^e01989^/+.* H, *GMR-Gal4:UAS-MeCP2^FLM119-2M^/+; UAS-trc^LD^/+.* I, *GMR-Gal4:UAS-MeCP2^FLM119-2M^/+; trx^KG04195^/+.* J, *GMR-Gal4/+.* K, *GMR-Gal4:UAS-MeCP2^FLM119-2M^/+.* L, *GMR-Gal4:UAS-MeCP2^FLM119-2M^/+; UAS-Scm/+.* M, *GMR-Gal4/+.* N, *GMR-Gal4:UAS-MeCP2^FLM119-2M^/+.* O, *GMR-Gal4:UAS-MeCP2^FLM119-2M^/UAS-osa.* P, *GMR-Gal4/UAS-osa.* Q, *GMR-Gal4:UAS-MeCP2^FLM119-2M^/UAS-pbl.* R, *GMR-Gal4/UAS-pbl.*

Each mutant line carrying a candidate modifier was crossed to flies expressing the full-length MeCP2 allele from the *GMR-Gal4* driver and screened for both enhancers and suppressors. The initial hits in this screen were then re-evaluated with an independent full-length MeCP2 transgenic line. Genes that modify the MeCP2 phenotypes across multiple strains and MeCP2 lines are the chromatin remodeling genes *Additional sex combs* (*Asx*), *corto*, *osa*, *Sex combs on midleg* (*Scm*), and *trithorax (trx)*, the kinase *tricornered (trc)* and the UBE3A target *pebble (pbl)* (Table 1). Partial loss of function of *Asx*, *corto*, *osa*, *pebble*, or *Scm* suppress the eye phenotype induced by full-length MeCP2, while *trc* has a similar effect when it is overexpressed (Figure 4A–H, note improved ommatidial organization relative to MeCP2 control). In contrast, enhancement of the eye phenotype was observed in MeCP2 animals with either loss-of-function mutations in *trx* or overexpression alleles of *Scm*, *osa*, and *pbl* (Figure 4I-4R, Figure S1, note greater ommatidial disruption, loss of interommatidial bristles and, in some cases, reduction in eye size and eye depigmentation). To exclude the possibility that modifiers of the Gal4-UAS system may simply cause changes in expression of MeCP2, western blot analysis was performed and demonstrated that the modifiers did not alter the level of MeCP2 protein (Figure S2).

Each modifier line found to alter the full-length MeCP2 phenotype was also investigated in the context of the Δ166 and R294X MeCP2 alleles to determine if the modification was dependent upon a specific MeCP2 domain. For the MeCP2 Δ166 allele, all genetic modifiers behaved similarly to the full-length MeCP2 allele (Table 1, Figure S3).

Since the MeCP2 R294X allele does not dramatically alter the structure of the eye, suppression was assessed primarily by gain in the amount of eye pigmentation. Enhancement was assessed by increased loss of pigmentation and/or disruption in the external structure of the eye. We found similar phenotype modifications as with full-length MeCP2 with two interesting exceptions (Table 1 and Figure S4). Partial loss of *Sin3A* function, which enhances full-length MeCP2 (compare Figures 3B and 3C), suppresses MeCP2 R294X phenotypes (compare Figures S4D and S4A). Partial loss of *trx* function, which enhances the full-length MeCP2 phenotype (compare Figures 4B and 4I), but, in the case of the *trx^E2^* allele suppresses the R294X phenotype (compare Figures S4A and S4P).

**Figure 5:**
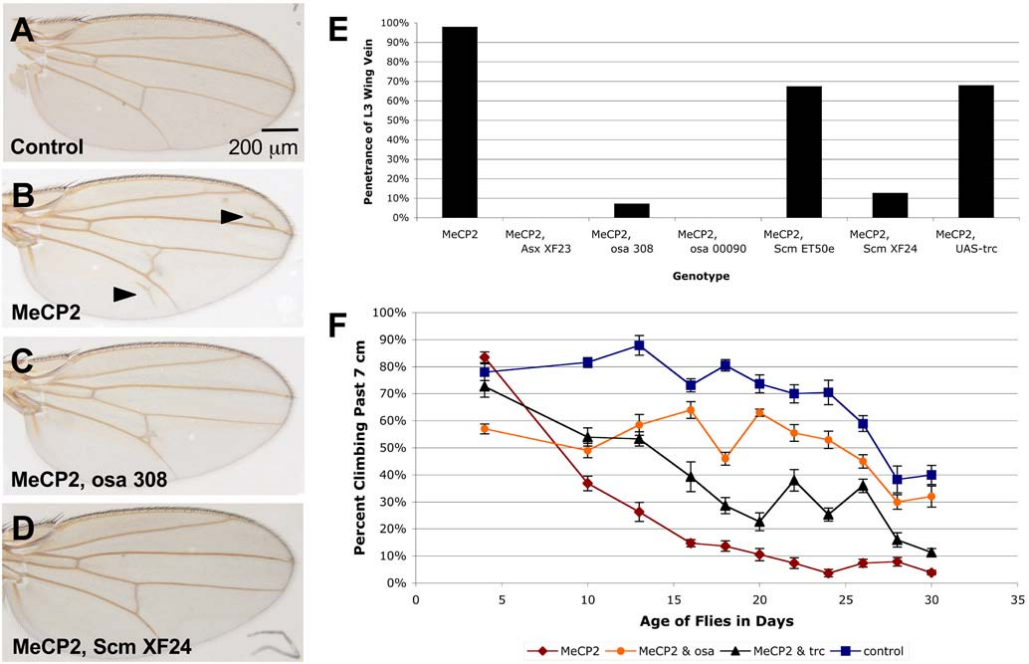
Genetic modifiers of the MeCP2 eye phenotype also suppress the L3 wing vein phenotype, and the motor impairment caused by neuronal overexpression of MeCP2. A–B. Expression of MeCP2 in the wing pouch by the *C5-Gal4* driver causes extra wing vein tissue (arrowheads near L3 and L5 veins) as compared to control flies. C–D. This phenotype is suppressed by genetic modifiers of the external eye phenotype including osa and Scm. E. Quantification of the L3 wing vein phenotype demonstrates that alleles of Asx, osa, Scm, and trc are all able to significantly suppress the wing vein phenotype (p<0.05 in all cases). F. Overexpression of full-length MeCP2 by the neuronal driver *CHA-Gal4* leads to a motor function impairment as measured in a climbing assay that becomes more severe over time. When MeCP2 is expressed in the presence of either a loss-of-function osa^00090^ allele or the gain of function UAS-trc^LD^ allele, the severity of the climbing phenotype is reduced (Repeated measures ANOVA p<0.001 at day 13 for osa^00090^, and for UAS-trc^LD^ at day 10). Each sample represents an initial group of 20 virgin female flies except one control group which had 15 virgin flies. Error bars represent the standard error. Experiment was performed in duplicate yielding similar results, but only one data set is shown.

The candidate suppressor genes were then further tested against the full-length MeCP2 allele in a second independent assay using the L3 wing vein phenotype (Figure 5A–E). Indeed, alleles of *Asx*, *osa*, *Scm* and *trc* are able to decrease the penetrance of the L3 wing vein phenotype. Furthermore, loss of function of *osa* and overexpression of *trc* improve the climbing phenotype caused by neuronal-specific expression of MeCP2 (Figure 5F). Consistent modification of MeCP2 phenotypes in different tissues, including a behavioral phenotype caused by neural-specific expression, provides additional evidence for the capacity of theses genes to modulate MeCP2 function.

## Discussion

We have used the *Drosophila* model system to facilitate the identification of genes capable of counterbalancing the consequences of altered levels of the human MeCP2 protein. First, we established anatomical and behavioral assays to assess the effects of expressing human MeCP2 in flies. We used an eye phenotype as a primary assay for the genetic screen, and impaired motor performance and other phenotypes as secondary assays for validating purposes. The eye phenotype has been used successfully in a variety of genetic screens including screens for enhancer/suppressors of other neurological disease models. Although expression of a variety of “toxic” human proteins leads to apparently similar “rough” eye phenotypes, their specificity is demonstrated when comparing the genetic modifiers uncovered in the screens. For example, there is little or no overlap between the MeCP2 modifiers reported here and modifiers of the eye phenotype produced by expression of ataxin-1 [32],[33] or huntingtin [27]. In contrast, we found that the majority of the modifier genes modulating the eye phenotype caused by wild-type MeCP2 similarly modulate the phenotypes caused by the R294X and Δ166 MeCP2 mutations. Two exceptions are *Sin3A* and *trx*, which have opposite effects on wild-type and R294X MeCP2 (Table 1, Figures 3B, C versus Figures S4A, D, and Figures 4B, I versus Figures S4A, P). MeCP2 associates with a co-repressor complex containing Sin3A through the TRD domain [14], which is partially deleted in the truncated R294X protein. This mutant also lacks the MeCP2 C-terminal region that is important for interactions with chromatin in vitro [34]. The TRD domain and/or C-terminal region may thus be involved in the observed genetic interaction between MeCP2 and *trx*. It is important to note that both *Sin3A* and *trx* do modify the eye phenotype of R294X MeCP2 animals, albeit in the opposite way from the wild-type MeCP2. Thus, the TRD/C-terminal domains may play a modulating role rather than being required for the interaction.

A commonly accepted model of MeCP2 function postulates that MeCP2 binds to methylated CpG islands in promoters where it recruits histone deacetylases and other co-repressors to silence gene transcription [14],[35]. However, accumulating evidence suggests that this may be too simple a view of MeCP2 function. For example, MeCP2 binds to unmethylated DNA with affinity only 3 times weaker than to methylated DNA [36], and MeCP2 also binds [37] or requires AT sequences for binding [38]. Moreover, MeCP2 interacts with both methylated and unmethylated chromatin and leads to alterations in the secondary structure of both types of chromatin [34],[39]. In addition, large-scale mapping of MeCP2 binding sites in chromosomal regions containing candidate MeCP2 target genes revealed that: 1) MeCP2 is absent from highly methylated promoters, 2) only ∼6% of MeCP2 binding sites are in CpG islands, and 3) many MeCP2-bound promoters are actively expressed [40]. Furthermore, a recent study of gene expression patterns in mice that either lack or overexpress MeCP2 suggests that many genes are activated by MeCP2 [41]. Here we show that the methyl-CpG-binding domain is not necessary for association of the MeCP2 protein with chromatin in polytene chromosomes (Figures 1H-H”), nor is it required to produce an eye phenotype in *Drosophila* (Figures 2D-D”). In this context it is interesting to note that unlike mammals, bacteria, plants, and other insects, the levels of DNA methylation are very low in *Drosophila* [42]. Together these data suggest that MeCP2 function may be more complex than previously thought. MeCP2 may regulate both methylated and unmethylated target genes in vivo, possibly as part of large protein complex(es) of chromatin remodelling proteins regulating gene expression both positively and negatively.

Using a candidate gene approach, we provide proof of principle that modulating the activity of modifier genes can amend MeCP2 function in vivo. Among this group of genes is the kinase *trc*, a member of the NDR (nuclear Dbf-related) family. We could not detect alterations in the phosphorylation of MeCP2 in *trc* mutants (data not shown). However, there is evidence that both *trc* and one of its mammalian homologs, NDR2, are involved in dendritic formation [43],[44], a feature also found to be affected by mutations in MeCP2. Also, modification of the MeCP2 phenotype by the E3 ligase UBE3A target *pbl* [31] is noteworthy due to the similarities between Rett and Angelman syndromes. Patients with Angelman-like features have been identified with MeCP2 mutations [45],[46] and, while still controversial, some studies have demonstrated a decrease of UBE3A in Rett patients and Mecp2 null mice [47]–[49]. The data presented here suggest that shared pathways may be involved in Rett and Angelman syndromes.

Misregulation of neuronal genes caused by alterations in MeCP2 activity is thought to cause Rett and Rett-like syndromes [50],[51]. One possible avenue for therapy is to identify the MeCP2 target genes misregulated during disease and to restore their normal regulation. This approach may prove impractical if the targets are numerous or difficult to identify due to subtle variations in expression levels in response to MeCP2 activity [52]–[57]. A possible future treatment based on gene therapy to restore normal levels of MeCP2 also seems improbable. The nervous systems of Rett patients are mosaic due to random X-chromosome inactivation causing some neurons expressing the normal while others expressing the mutant allele. Therefore, in the context of neurons expressing the wild-type allele, gene therapy is not possible because doubling of MeCP2 also leads to disease [5],[6],[58]. An alternative approach is to identify molecular mechanisms capable of compensating for the misregulation of target genes caused by MeCP2 altered levels. This study provides support for the validity of this approach. We identified specific chromatin remodeling genes of the Pc-G and Trx-G (i.e., *Asx, corto, osa,* and *Scm*) that suppress the phenotypes caused by MeCP2 overexpression in *Drosophila*. Interestingly, both in *Drosophila* and mammals, mutations in genes of either Pc-G or Trx-G also suppress the body patterning abnormalities caused by mutations in members of the other group [17],[19].

In conclusion, human MeCP2 protein expressed in *Drosophila* maintains important features observed in mammals such as phosphorylation and association with the chromatin. The novel modifiers identified in this model system point to potential therapeutic targets that might be more amenable to manipulation than MeCP2, and thus they provide new opportunities to develop therapies for Rett syndrome and related neurological disorders.

## Methods

### Generation of MeCP2 Constructs and *Drosophila* Lines

Each of the MeCP2 alleles described was cloned into the pUAST vector in order to utilizing the GAL4-UAS system (Figure 1A). The full-length human cDNA of the *MECP2_e2* isoform (1461 nucleotides, 486 amino acids) was subcloned into the EcoRI site of the pUAST vector. The remaining three alleles were generated by PCR mutagenesis of this initial construct. MeCP2 R294X was amplified with primers that attached a stop codon and Kpn I site to the C-terminal end. This PCR fragment was digested with EcoRI and Kpn I and then ligated between these restriction sites in pUAST. Primers amplifying the MeCP2 Δ166 fragment added an EcoRI site, a conserved *Drosophila* consensus sequence (TCGAC), and an ATG start site to the N-terminal side of the protein. Transgenic *Drosophila* lines were generated by injection of these constructs in embryos following standard methods. We generated eleven MeCP2 full-length lines, ten MeCP2 R106W lines, three MeCP2 R294X lines and ten MeCP2 Δ166 lines. Additional *Drosophila* lines were obtained from the Bloomington *Drosophila* Stock Center, the Harvard Medical School Exelixis *Drosophila* Stock Collection, and private investigators (see acknowledgements).

### Western Blot Analysis and Alkaline Phosphatase Treatment

Protein was collected from *Drosophila* heads in a solution of 5% β-mercaptoethanol in Laemmli Sample Buffer (Bio-Rad). For the alkaline phosphatase treatment, *Drosophila* heads were collected in protein extraction buffer (PBS with 0.1% Nonident P40 and protease inhibitors), samples were smashed and kept on ice for 1 hour, vortexing each 10 minutes in order to facilitate protein extraction. Samples were then mixed 1∶1 with the calf intestinal alkaline phosphatase (CIP) treatment, 10% CIP enzyme (New England Biolabs), 30% CIP buffer in water, and incubated for 50 minutes at 37°C. Laemmli Sample Buffer was then added to these reactions. Proteins were run on SDS-PAGE gels with eight *Drosophila* heads per lane (except for the alkaline phosphatase experiment which had ten heads per lane). Proteins were then transferred to nitrocellulose membrane (Optitran) using 10mM CAPS with 10% methanol. Membranes were blocked in BLOTTO 5% Non-Fat Dry Milk (Bio-Rad) in TBS-T (100mM Tris-Cl pH 7.5, 150mM NaCl, 0.1% Tween 20). The following antibodies were used diluted in BLOTTO: anti-MeCP2 antibodies (1∶1000, Upstate, #07-013, and Affinity, #PA1-887), anti-lamin C (1∶1000, Developmental Studies Hybridoma Bank, #LC28.26), and anti-tubulin (1∶5000, Developmental Studies Hybridoma Bank, #E7). Anti-phosphorylated MeCP2 S423 was diluted in 5% BSA (1∶1000) [25]. Anti-rabbit and anti-mouse horseradish peroxidase-conjugated secondary antibodies (Bio-Rad) were diluted 1∶5000 in BLOTTO and membranes were developed using chemiluminescence with either the ECL kit (Amersham Biosciences) or the SuperSignal West Dura kit (Pierce). Quantification of western blots was performed on a densitometer (Molecular Dynamics) using the ImagQuant program.

### Scanning Electron Microscopy

Experimental and control lines were crossed to flies with the eye specific *GMR-Gal4* driver. Offspring were sorted by genotype and whole adult flies were sequentially dehydrated in ethanol, critically-point dried and placed on aluminum mounts. Samples were coated with a platinum alloy for a thickness of 50 nm and flash carbon coated. *Drosophila* heads were then analyzed with a JEOL JSM-5900 scanning electron microscope.

### L3 Wing Vein Assays

Experimental and control lines were crossed to flies with the *C5-Gal4* driver and cultured at 25°C. Once the offspring had eclosed, flies were sorted by genotype and each individual wing was scored under a light microscope for extra vein tissue near or attached to the L3 wing vein. Wings were removed from flies and mounted in DPX Mounting Medium (Electron Microscopy Sciences).

### Climbing Assay

Experimental and control lines were crossed to flies with the *CHA-Gal4* driver and cultured at 25°C. Virgins were collected of each genotype and sorted into batches of 20 flies. Flies were enclosed inside two clean, unused 9.25 cm culturing tubes that had been taped together, for a total height of 18.5 cm. Flies were tapped down to the bottom of the vial and permitted 18 seconds to climb within both tubes to the top. At the end of 18 seconds, flies were scored as to whether their final position was either above or below 7 cm. Each group was trained in this procedure for 10 trials and then tested for 10 trials. Trials were performed between 3–6 pm.

### Immunofluorescence Staining of Polytene Chromosomes

Experimental and control lines were crossed to flies with the ubiquitous *Actin5c-Gal4* driver and cultured at 25°C. Salivary glands were dissected from third instar larvae, fixed with formaldehyde, and squashed according to standard protocols. Samples were blocked with PBT with 0.2% BSA and 5% horse serum to reduce background. Primary antibodies for MeCP2 were used (1∶100, Affinity and 1∶200, Upstate). The secondary immunofluorescence goat anti-rabbit Cy3 antibody was used at a 1∶200 dilution. The slides were also treated with an RNase cocktail (1∶1000, Ambion) and then TOTO-3 (1∶2000, Molecular Probes) to stain the DNA for confocal microscopy. Slides were then mounted with a drop of Vectashield containing DAPI in order to visualize the DNA by eye. Images were collected by confocal microscopy using the AxioVision and ImageJ programs.

## Supporting Information

Figure S1 Overexpression of the novel genetic modifier osa enhances the MeCP2 external eye phenotype. MeCP2 driven by *GMR-Gal4* at 27.5°C causes increased disorganization of the ommatidia and interommatidial bristles compared to controls (A–B). This disorganization is increased when combined with an overexpression allele of the chromatin remodeling gene *osa* such that the overall size of the eye is smaller, ommatidia are indistinguishable, there are no interommatidial bristles, and necrotic spots are visible, as shown with arrow (C). Overexpression of osa alone by *GMR-Gal4* also disrupts the external eye structure (D), but to a much milder degree as compared to co-expression of MeCP2 and osa. Genotypes: A, *GMR-Gal4/+.* B, *GMR-Gal4:UAS-MeCP2^FLM119-2M^/+.* C, *GMR-Gal4:UAS-MeCP2^FLM119-2M^/UAS-osa.* D, *GMR-Gal4/UAS-osa*.
(3.3 MB TIF)

Figure S2 Genetic modifiers do not alter protein levels of whole MeCP2 or phosphorylated S423 MeCP2. Western blots were performed using the heads of flies expressing MeCP2 by *GMR-Gal4* in the presence of modifiers involved in chromatin remodeling (A) and kinases (B). Key genetic modifiers that were found to be the most consistently validated in the external eye assay and secondary assays are marked with an asterisk. All modifiers were compared to the two MeCP2 positive controls on the same blot. Quantification by densitometry failed to find a significant alteration of MeCP2 levels in the case of modifiers as compared to the variation in the internal positive controls.
(1.9 MB TIF)

Figure S3 Genetic modifiers of the full-length MeCP2 eye phenotype modify MeCP2 Δ166 in a similar manner. Light microscopy images of the external eye of *Drosophila* expressing the MeCP2 Δ166 allele and the indicated modifier genes. Control is shown in A. All flies cultured at 29°C. Genotypes: A, *GMR-Gal4:UAS-MeCP2 Δ166/+*. B, *GMR-Gal4: UAS-MeCP2 Δ166/Sin3A^dQ4^.* C, *GMR-Gal4: UAS-MeCP2 Δ166/crol^e0407^.* D, *Smr^e04389^/+; GMR-Gal4: UAS-MeCP2 Δ166/+*. E, *GMR-Gal4:UAS-MeCP2 Δ166/UAS-osa*. F, *GMR-Gal4:UAS-MeCP2 Δ166/UAS-pbl.* G, *GMR-Gal4:UAS-MeCP2 Δ166/+; UAS-Scm/+*. H, *GMR-Gal4:UAS-MeCP2 Δ166/+; trx^E2^/+.* I, *GMR-Gal4:UAS-MeCP2 Δ166/Asx^XF23^.* J, *GMR-Gal4:UAS-MeCP2 Δ166/+; corto^c03244^/+.* K, *GMR-Gal4:UAS-MeCP2 Δ166/+; osa^00090^/+.* L, *GMR-Gal4:UAS-MeCP2 Δ166/+; UAS-trc^LD^/+.* M, *GMR-Gal4:UAS-MeCP2 Δ166/+; pbl^09645^/+.* N, *GMR-Gal4:UAS-MeCP2 Δ166/+; Scm^e01989^/+*.
(9.8 MB TIF)

Figure S4 Genetic modifiers of the full-length MeCP2 eye phenotype in the context of the MeCP2 R294X allele. Light microscopy images of the external eye of *Drosophila* expressing the MeCP2 R294X allele and the indicated modifier genes. Control is shown in A. All flies cultured at 27.5°C. Note that partial loss of function of Sin3A (panel D) suppresses the MeCP2 R294X phenotype shown in A, while it enhances the full-length MeCP2 eye phenotype (see Figures 3B and 3C). Likewise, the *trx^E2^* allele suppresses the MeCP2 R294X phenotype (compare panels A and P), while it enhances the full-length MeCP2 eye phenotype (see Figures 4B and 3I). All other genetic modifiers have similar effects on full-length MeCP2 and MeCP2 R294X phenotypes, although the genetic interactions may be evident with some but not all alleles of each modifier genes (see Table 1). Genotypes: A, *GMR-Gal4:UAS-MeCP2 R294X/+*. B, *GMR-Gal4: UAS-MeCP2 R294X/crol^c04670^.* C, *Smr^e04377^/+; GMR-Gal4: UAS-MeCP2 R294X/+*. D,*GMR-Gal4: UAS-MeCP2 R294X/Sin3A^dQ4^.* E, *GMR-Gal4:UAS-MeCP2 R294X/Asx^XF23^.* F, *GMR-Gal4:UAS-MeCP2 R294X/+; corto^c03244^/+.* G, *GMR-Gal4:UAS-MeCP2 R294X/+; osa^00090^/+.* H, *GMR-Gal4:UAS-MeCP2 R294X/+; pbl^5^/+.* I, *GMR-Gal4:UAS-MeCP2 R294X/+; UAS-trc^LD^/+.* J, *GMR-Gal4:UAS-MeCP2 R294X/Asx^1^*. K, *GMR-Gal4:UAS-MeCP2 R294X/UAS-osa*. L, *GMR-Gal4:UAS-MeCP2 R294X/UAS-pbl.* M, *GMR-Gal4:UAS-MeCP2 R294X/+; pbl^09645^/+.* N, *GMR-Gal4:UAS-MeCP2 R294X/+; Scm^ET50e^/+.* O, *GMR-Gal4:UAS-MeCP2 R294X/+; trx^E2^/+.* P, *GMR-Gal4:UAS-MeCP2 R294X/+; trx^1^/+.*
(8.5 MB TIF)

Table S1 *Drosophila* homologs of known MeCP2 Interactors.
(0.07 MB DOC)

Video S1 Neuronal specific overexpression of MeCP2 results in motor dysfunction. Flies aged 30 days after eclosion are shown in a climbing assay. After being tapped to the bottom of the vial, flies are given 18 seconds to climb past 7 cm, marked by a line on the tube. The control flies (left side, blue) show good climbing ability, while MeCP2 transgenic flies (right side, red) do not perform well.
(3.0 MB MOV)
