# Supplementary figures and images for: Genetic Modifiers of MeCP2 Function in Drosophila
Source: PLoS Genet. 2008 Sep 5;4(9):e1000179. doi: 10.1371/journal.pgen.1000179 (PMC2518867; doi:10.1371/journal.pgen.1000179)

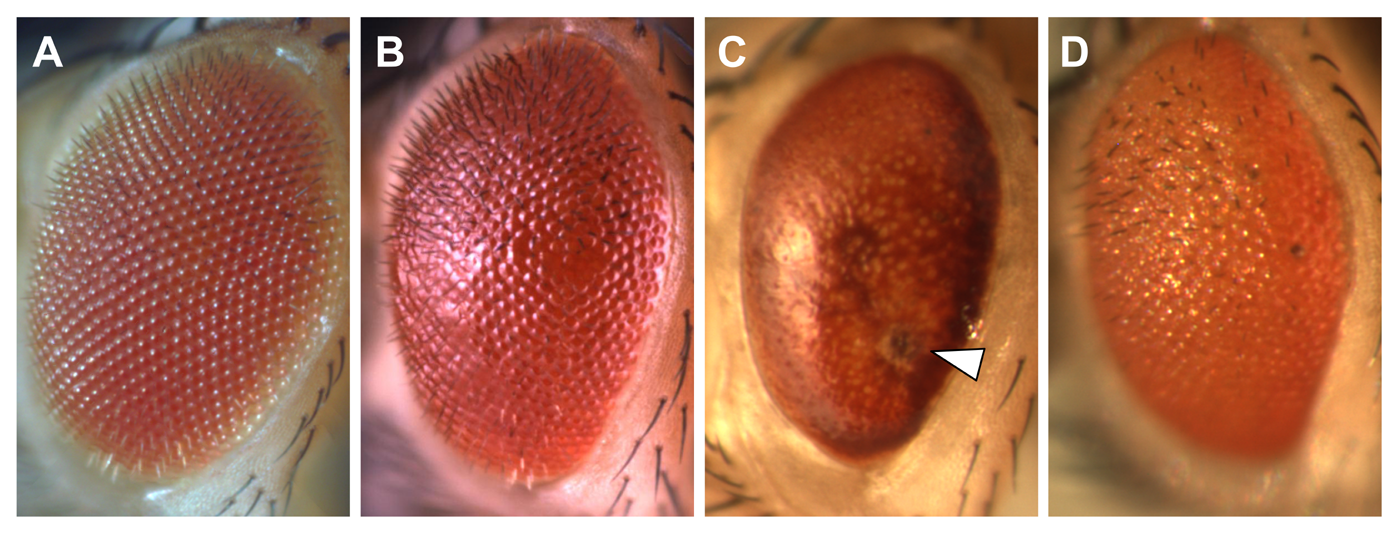

Supplement: Figure S1 — Overexpression of the novel genetic modifier osa enhances the MeCP2 external eye phenotype. MeCP2 driven by GMR-Gal4 at 27.5°C causes increased disorganization of the ommatidia and interommatidial bristles compared to controls (A–B). This disorganization is increased when combined with an overexpression allele of the chromatin remodeling gene osa such that the overall size of the eye is smaller, ommatidia are indistinguishable, there are no interommatidial bristles, and necrotic spots are visible, as shown with arrow (C). Overexpression of osa alone by GMR-Gal4 also disrupts the external eye structure (D), but to a much milder degree as compared to co-expression of MeCP2 and osa. Genotypes: A, GMR-Gal4/+. B, GMR-Gal4:UAS-MeCP2FLM119-2M/+. C, GMR-Gal4:UAS-MeCP2FLM119-2M/UAS-osa. D, GMR-Gal4/UAS-osa. (3.3 MB TIF) [file pgen.1000179.s001.tif]

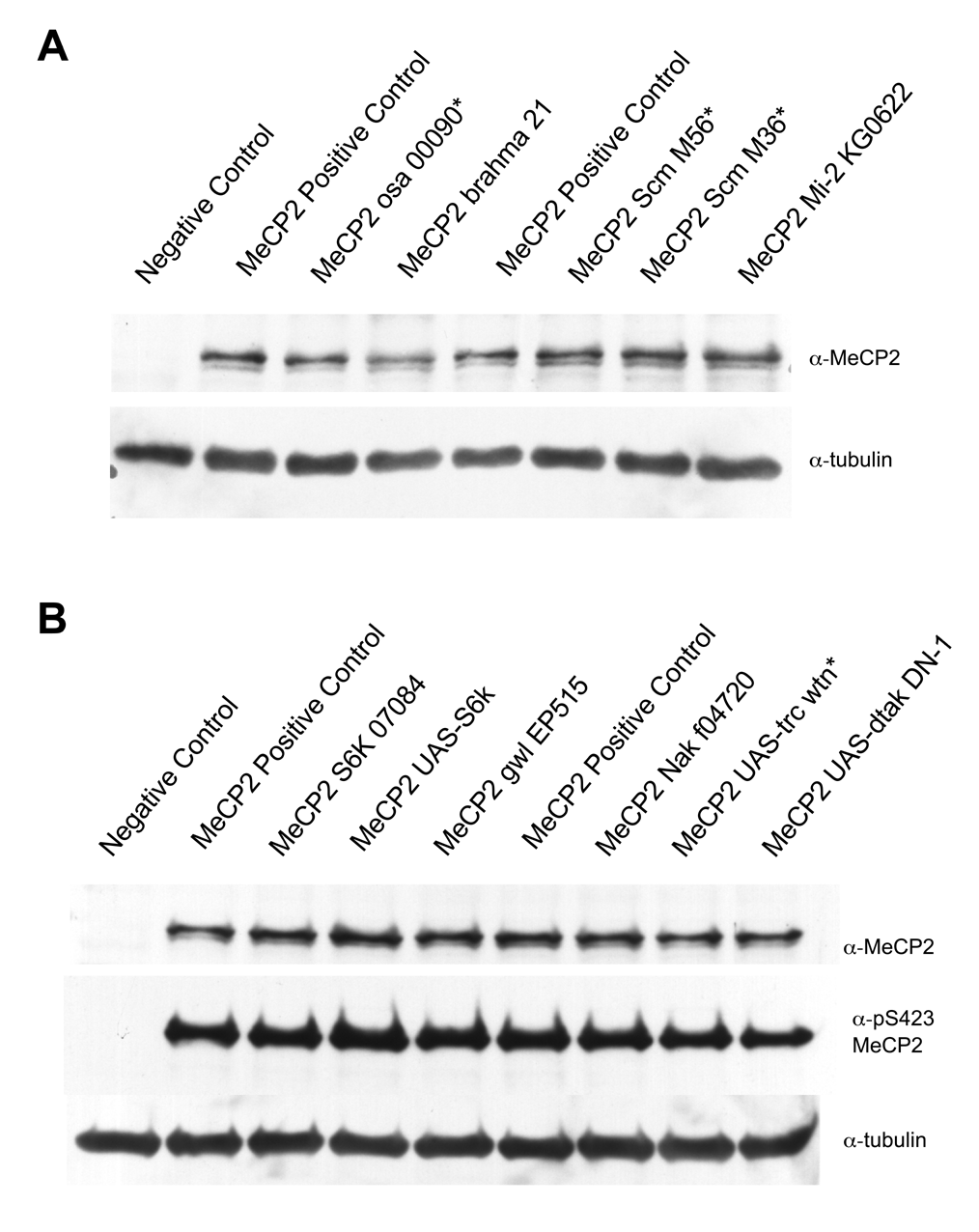

Supplement: Figure S2 — Genetic modifiers do not alter protein levels of whole MeCP2 or phosphorylated S423 MeCP2. Western blots were performed using the heads of flies expressing MeCP2 by GMR-Gal4 in the presence of modifiers involved in chromatin remodeling (A) and kinases (B). Key genetic modifiers that were found to be the most consistently validated in the external eye assay and secondary assays are marked with an asterisk. All modifiers were compared to the two MeCP2 positive controls on the same blot. Quantification by densitometry failed to find a significant alteration of MeCP2 levels in the case of modifiers as compared to the variation in the internal positive controls. (1.9 MB TIF) [file pgen.1000179.s002.tif]

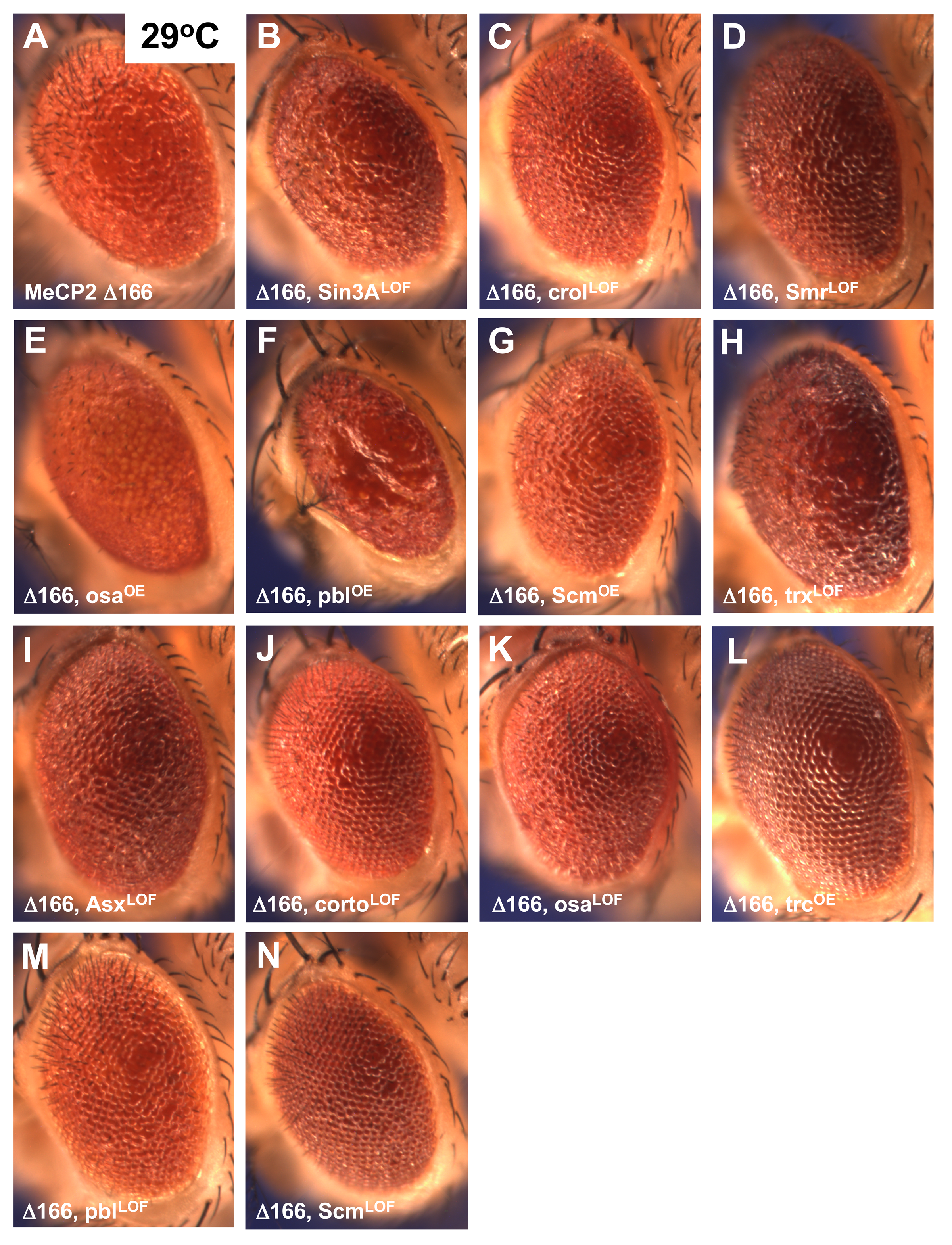

Supplement: Figure S3 — Genetic modifiers of the full-length MeCP2 eye phenotype modify MeCP2 Δ166 in a similar manner. Light microscopy images of the external eye of Drosophila expressing the MeCP2 Δ166 allele and the indicated modifier genes. Control is shown in A. All flies cultured at 29°C. Genotypes: A, GMR-Gal4:UAS-MeCP2 Δ166/+. B, GMR-Gal4: UAS-MeCP2 Δ166/Sin3AdQ4. C, GMR-Gal4: UAS-MeCP2 Δ166/crole0407. D, Smre04389/+; GMR-Gal4: UAS-MeCP2 Δ166/+. E, GMR-Gal4:UAS-MeCP2 Δ166/UAS-osa. F, GMR-Gal4:UAS-MeCP2 Δ166/UAS-pbl. G, GMR-Gal4:UAS-MeCP2 Δ166/+; UAS-Scm/+. H, GMR-Gal4:UAS-MeCP2 Δ166/+; trxE2/+. I, GMR-Gal4:UAS-MeCP2 Δ166/AsxXF23. J, GMR-Gal4:UAS-MeCP2 Δ166/+; cortoc03244/+. K, GMR-Gal4:UAS-MeCP2 Δ166/+; osa00090/+. L, GMR-Gal4:UAS-MeCP2 Δ166/+; UAS-trcLD/+. M, GMR-Gal4:UAS-MeCP2 Δ166/+; pbl09645/+. N, GMR-Gal4:UAS-MeCP2 Δ166/+; Scme01989/+. (9.8 MB TIF) [file pgen.1000179.s003.tif]

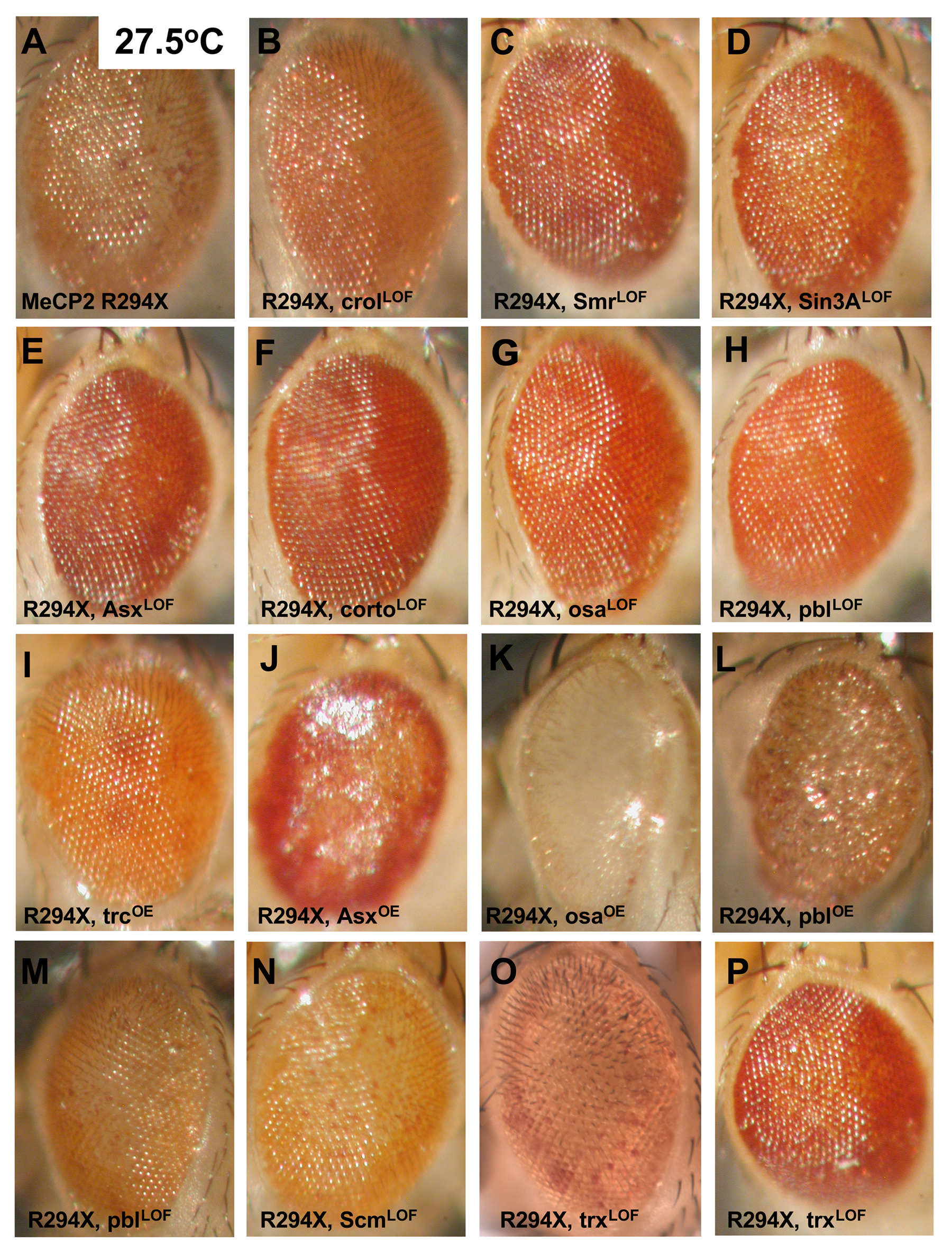

Supplement: Figure S4 — Genetic modifiers of the full-length MeCP2 eye phenotype in the context of the MeCP2 R294X allele. Light microscopy images of the external eye of Drosophila expressing the MeCP2 R294X allele and the indicated modifier genes. Control is shown in A. All flies cultured at 27.5°C. Note that partial loss of function of Sin3A (panel D) suppresses the MeCP2 R294X phenotype shown in A, while it enhances the full-length MeCP2 eye phenotype (see Figures 3B and 3C). Likewise, the trxE2 allele suppresses the MeCP2 R294X phenotype (compare panels A and P), while it enhances the full-length MeCP2 eye phenotype (see Figures 4B and 3I). All other genetic modifiers have similar effects on full-length MeCP2 and MeCP2 R294X phenotypes, although the genetic interactions may be evident with some but not all alleles of each modifier genes (see Table 1). Genotypes: A, GMR-Gal4:UAS-MeCP2 R294X/+. B, GMR-Gal4: UAS-MeCP2 R294X/crolc04670. C, Smre04377/+; GMR-Gal4: UAS-MeCP2 R294X/+. D,GMR-Gal4: UAS-MeCP2 R294X/Sin3AdQ4. E, GMR-Gal4:UAS-MeCP2 R294X/AsxXF23. F, GMR-Gal4:UAS-MeCP2 R294X/+; cortoc03244/+. G, GMR-Gal4:UAS-MeCP2 R294X/+; osa00090/+. H, GMR-Gal4:UAS-MeCP2 R294X/+; pbl5/+. I, GMR-Gal4:UAS-MeCP2 R294X/+; UAS-trcLD/+. J, GMR-Gal4:UAS-MeCP2 R294X/Asx1. K, GMR-Gal4:UAS-MeCP2 R294X/UAS-osa. L, GMR-Gal4:UAS-MeCP2 R294X/UAS-pbl. M, GMR-Gal4:UAS-MeCP2 R294X/+; pbl09645/+. N, GMR-Gal4:UAS-MeCP2 R294X/+; ScmET50e/+. O, GMR-Gal4:UAS-MeCP2 R294X/+; trxE2/+. P, GMR-Gal4:UAS-MeCP2 R294X/+; trx1/+. (8.5 MB TIF) [file pgen.1000179.s004.tif]
