# Supplementary material for: Genetic Modifiers of MeCP2 Function in Drosophila
Source: PLoS Genet. 2008 Sep 5;4(9):e1000179. doi: 10.1371/journal.pgen.1000179 (PMC2518867; doi:10.1371/journal.pgen.1000179)
Supplement: Table S1 — Drosophila homologs of known MeCP2 Interactors. (0.07 MB DOC) [file pgen.1000179.s005.doc]

| **Supplemental Table 1.** *Drosophila* homologs of known MeCP2 Interactors | | | | | |
| --- | --- | --- | --- | --- | --- |
|  |  |  |  |  |  |
|  |  |  | **region of highest homology** | | |
| **Human Protein** Closest Related *Drosophila* Protein | protein total length in amino acids | BLAST E Value | length in amino acids | percent of identity | percent of similarity |
|  |  |  |  |  |  |
| **Brahma/Smarca2** | **1583** |  |  |  |  |
| Brahma | 1638 | 0.E+00 | 1566 | 52% | 64% |
|  |  |  |  |  |  |
| **C-ski** | **728** |  |  |  |  |
| snoN | 338 | 4.E-56 | 243 | 48% | 60% |
|  |  |  |  |  |  |
| **HDAC1** | **482** |  |  |  |  |
| Rpd3 | 512 | 0.E+00 | 471 | 77% | 88% |
|  |  |  |  |  |  |
| **N-CoR** | **2440** |  |  |  |  |
| Smrter | 3604 | 2.E-72 | 651 | 30% | 48% |
|  |  |  |  |  |  |
| **REST** | **1097** |  |  |  |  |
| crooked legs | 891 | 4.E-36 | 269 | 28% | 46% |
|  |  |  |  |  |  |
| **Sin3A** | **1273** |  |  |  |  |
| Sin3A | 1776 | 3.E-147 | 886 | 39% | 52% |
|  |  |  |  |  |  |
| **UBE3A/E6-AP** | **875** |  |  |  |  |
| Ube3a/As | 973 | 0.E+00 | 956 | 41% | 58% |
|  |  |  |  |  |  |
| **YB-1** | **324** |  |  |  |  |
| ypsilon schachtel | 352 | 1.E-47 | 313 | 39% | 52% |
|  |  |  |  |  |  |
